# Actions of Agonists and Antagonists of the ghrelin/GHS-R Pathway on GH Secretion, Appetite, and cFos Activity

**DOI:** 10.3389/fendo.2013.00025

**Published:** 2013-03-18

**Authors:** Rim Hassouna, Alexandra Labarthe, Philippe Zizzari, Catherine Videau, Michael Culler, Jacques Epelbaum, Virginie Tolle

**Affiliations:** ^1^UMR-S 894 INSERM, Centre de Psychiatrie et Neurosciences, Université Paris Descartes, Sorbonne Paris CitéParis, France; ^2^IPSENMilford, MA, USA

**Keywords:** ghrelin, GHS-R, BIM-28163, BIM-28131, antagonist, food intake, GH secretion, cFos

## Abstract

The stimulatory effects of ghrelin, a 28-AA acylated peptide originally isolated from stomach, on growth hormone (GH) secretion and feeding are exclusively mediated through the growth hormone secretagogue 1a receptor (GHS-R1a), the only ghrelin receptor described so far. Several GHS-R1a agonists and antagonists have been developed to treat metabolic or nutritional disorders but their mechanisms of action in the central nervous system remain poorly understood. In the present study, we compared the activity of BIM-28163, a GHS-R1a antagonist, and of several agonists, including native ghrelin and the potent synthetic agonist, BIM-28131, to modulate food intake, GH secretion, and cFos activity in arcuate nucleus (ArcN), nucleus tractus solitarius (NTS), and area postrema (AP) in wild-type and NPY-GFP mice. BIM-28131 was as effective as ghrelin in stimulating GH secretion, but more active than ghrelin in inducing feeding. It stimulated cFos activity similarly to ghrelin in the NTS and AP but was more powerful in the ArcN, suggesting that the super-agonist activity of BIM-28131 is mostly mediated in the ArcN. BIM-28163 antagonized ghrelin-induced GH secretion but not ghrelin-induced food consumption and cFos activation, rather it stimulated food intake and cFos activity without affecting GH secretion. The level of cFos activation was dependent on the region considered: BIM-28163 was as active as ghrelin in the NTS, but less active in the ArcN and AP. All compounds also induced cFos immunoreactivity in ArcN NPY neurons but BIM-28131 was the most active. In conclusion, these data demonstrate that two peptide analogs of ghrelin, BIM-28163, and BIM-28131, are powerful stimulators of appetite in mice, acting through pathways and key brain regions involved in the control of appetite that are only partially superimposable from those activated by ghrelin. A better understanding of the molecular pathways activated by these compounds could be useful in devising future therapeutic applications, such as for cachexia and anorexia.

## Introduction

Ghrelin is a 28 amino acid acylated peptide originally discovered in stomach tissues by inverse pharmacology (Kojima et al., 1999) as the endogenous ligand for the growth hormone (GH) Secretagogue-1a Receptor (GHS-R1a), an orphan receptor cloned a few years earlier from pig pituitaries (Howard et al., 1996). GH secretagogues are a family of synthetic peptidic or non-peptidic compounds developed from the early 1980s to stimulate GH secretion (Bluet-Pajot et al., 2001). As anticipated, ghrelin was initially described for its ability to stimulate GH secretion in several species including rodents and humans (Kojima et al., 1999; Tolle et al., 2001) but it is still to date the only orexigenic hormone produced in the gastrointestinal tract (Tschop et al., 2000).

The actions of ghrelin on GH secretion and feeding require the addition of an eight-carbon fatty acid that is attached on a serine in position 3 by the enzyme ghrelin-*O*-acyl-transferase (GOAT) (Gutierrez et al., 2008; Yang et al., 2008). They are exclusively mediated through the GHS-R1a (Sun et al., 2004), the only ghrelin receptor described so far. GHS-R1a is highly expressed in the hypothalamus, a region involved in the control of GH secretion and appetite, and also in the brainstem that receives informations from gut vagal afferents (Guan et al., 1997; Katayama et al., 2000). Within the hypothalamus, NPY neurons in the arcuate nucleus (ArcN) express the GHS-R1a (Willesen et al., 1999; Tannenbaum et al., 2003), and are a well-characterized target for ghrelin actions (Tannenbaum et al., 2003; Chen et al., 2004). Nevertheless, several studies have suggested that certain ghrelin actions may be mediated through a receptor that has yet to be identified.

Several GHS-R1a antagonists have been developed to decipher the function of the ghrelin/GHS-R pathway in the regulation of feeding behavior and GH secretion (Asakawa et al., 2003; Okimura et al., 2003; Beck et al., 2004; Halem et al., 2004; Demange et al., 2007; Esler et al., 2007; Petersen et al., 2009; Costantini et al., 2011; Moulin et al., 2013). Although they have all been clearly shown to antagonize exogenous ghrelin actions on GH secretion both in *in vitro* systems and *in vivo*, their biological effects *per se* on several biological parameters are contradictory and their mechanisms of action in the central nervous system remain poorly understood. Indeed, intracerebroventricular injections of two antagonists [d-Lys3]-GHRP6, an analog of one of the synthetic GHS (Asakawa et al., 2003) or [d-Arg-1, d-Phe-5, d-Trp-7,9,Leu-11]-substance P, an analog of substance P (Petersen et al., 2009) induces suppression of feeding in mice. In contrast [d-Lys3]-GHRP6 has no action on spontaneous ultradian GH secretion (Okimura et al., 2003). But results are biased by the fact that [d-Lys3]-GHRP6 also binds to all melanocortin receptors (Schioth et al., 1997) and [d-Arg-1, d-Phe-5, d-Trp-7,9,Leu-11]-substance P also has full inverse agonist action *in vitro* and *in vivo* (Holst et al., 2003; Petersen et al., 2009). To date, the effects of JMV2810, another recently developed GHS-R1a antagonist, on spontaneous feeding or GH secretion have not been reported (Demange et al., 2007).

Only the full-length ghrelin analog, BIM-28163 (now called RM-28163), has been tested on both spontaneous GH secretion and food intake. Treatment with this selective GHS-R1a antagonist over 48 h in rats reduces pulsatile GH secretion (Zizzari et al., 2005). In contrast, it increases food intake and weight gain as effectively as ghrelin when administered at 5- to 10-fold higher doses than ghrelin (Halem et al., 2004, 2005). Interestingly, BIM-28163 induces cFos activation in the dorsomedial nucleus of the hypothalamus (DMH) while it acts as an antagonist in the ArcN of the hypothalamus. These data are corroborated by a more recent study also describing a stimulatory effect of another GHS-R1a antagonist, GSK1614343, on feeding in rats and dogs (Costantini et al., 2011). Altogether these data are intriguing and suggest the existence of an unknown pathway mediating the effects of these ghrelin antagonists on feeding through either the GHS-R1a or another unknown receptor. Understanding the mechanisms of action of this compound that differentially affect feeding and GH secretion can be of clinical interest.

In the present study, we compared the effect of BIM-28163, BIM-28131(Strassburg et al., 2008; Palus et al., 2011), native ghrelin, and the combination of BIM-28163 + ghrelin in the modulation of food intake, GH secretion, and cFos activity in fed mice. Our aim was to map changes in cFos activation in several key brain regions controlling appetite and/or GH secretion, including the hypothalamic ArcN, ventromedial nucleus of the hypothalamus (VMH), nucleus tractus solitarius (NTS), and area postrema (AP) of the brainstem. In addition, we used NPY-GFP mice to test whether the feeding effects of the analogs were mediated, like ghrelin, through the orexigenic NPY neurons.

## Materials and Methods

### Animals

About 18–25-week-old C57BL6/J male and female mice, obtained either from Charles River or from our own colony, were used for feeding experiments. About 18–25-week-old C57BL6/J or NPY-Renilla GFP transgenic male and female mice backcrossed on the C57BL6/J background and expressing Renilla GFP under the transcriptional control of the NPY genomic sequence (Van Den Pol et al., 2009) were used for cFos experiments. Mice were housed at constant temperature and humidity, with a fixed 12-h light/dark cycle (lights-on at 7.00 a.m.) and free access to food and water. In addition, the animals were handled weekly to minimize stress. All experiments were carried out in accordance with the European Communities Council Directive (86/609/EEC) and were approved by the animal experimentation committee of Paris Descartes University.

### Peptides

Native ghrelin, BIM-28131 (a small peptide ghrelin agonist), and BIM-28163 (a full-length ghrelin analog antagonist) were obtained from IPSEN (Milford, MA, USA). Peptides were dissolved in a vehicle containing 0.9% saline +0.25% of bovin serum albumin (BSA).

### Automated food intake monitoring

One week prior to the experiments, 18–25-week-old C57BL6/J male mice were individually housed and acclimatized to the automated drinking/feeding stations (TSE Systems, GmbH, Germany). Feeding behavior was recorded continuously by means of high precision sensors, attached to the top of the cage lids. Meal patterns were analyzed using the following definition: a meal consists of the consumption of 0.03 g of food separated from the next feeding episode by at least 10 min as previously described (Yu et al., 2009; Stengel et al., 2010; Wang et al., 2011). For each mouse, the meal number, the total meal size (g), and the total meal duration (min) were measured within 4 h following peptide injections.

Experiments were performed during the light phase (10.00 a.m.–11.00 a.m.) and carried out in a cross-over designed manner so that each mouse received all treatments randomly separated by two washout days. On each experimental day, mice were injected intraperitoneally (ip) with either vehicle (0.9% saline containing 0.25% BSA), native ghrelin (30 nmol), BIM-28131 (30 nmol), BIM-28163 (150 nmol), or native ghrelin (30 nmol) combined with BIM-28163 (150 nmol).

### cFos immunohistochemistry, feeding, and GH measurements

About 18–25-week-old male and female C57BL6/J and NPY-GFP mice were individually housed and had free access to food and water at the time of injections. Vehicle, native ghrelin (30 nmol), BIM-28131 (30 nmol), BIM-28163 (150 nmol), or native ghrelin (30 nmol) combined with BIM-28163 (150 nmol) were injected ip in the early light phase (9.00 a.m.–11.00 a.m.).

A pre-weighed amount of food was distributed in each cage at the time of injections and weighed 90 min later in order to confirm the effects of the treatments on food consumption and to correlate food intake to the number of activated cFos nuclei. 15 min following the injection, 4 μl of whole blood was withdrawn from the tail vein, homogenized in 116 μl of GH buffer (PBS, 0.05% Tween) for GH measurements. Whole blood GH concentrations were evaluated by EIA as previously described (Steyn et al., 2011).

The number of nuclei immunoreactive for cFos protein were quantified 90 min after ip injection of peptides to determine which brain regions were activated by the compounds. Mice were deeply anesthetized with pentobarbital (5.5 mg/30 g BW) and perfused through the ascending aorta with saline 0.9% for 1 min followed by 4% paraformaldehyde (PFA) in phosphate buffer 0.1 M (PB) for an additional 9 min. The brains were removed, post-fixed for 2 h in 4% PFA and cryoprotected in 30% sucrose for 2 days at 4°C. Brains were then frozen in 2-methyl-butane and sectioned in the coronal plane at a thickness of 25 μm using a freezing microtome (Frigomobile, Leica, Wetzlar, Germany).

For the detection of cFos protein expression, free-floating sections were processed for immunohistochemistry. Sections were incubated in blocking buffer (10% Normal Donkey Serum, 0.3% Triton X-100 in 0.1 M TBS) for 1 h at room temperature then incubated with rabbit cFos antibody (1:20000, Ab-5, Jackson Laboratories, West Grove, PA, USA) in 1% NDS, 0.3% Triton X-100 in 0.1 M TBS overnight at room temperature. Sections were then rinsed 4 × 10 min in 0.1 M TBS and incubated with Cy3 conjugated Donkey Anti-Rabbit antibody (1:800 DAR-Cy3, Jackson Laboratories, West Grove, PA, USA) 1 h at room temperature and then rinsed 4 × 10 min in 0.1 M TBS. Sections were mounted with fluoromount and quantified using a Zeiss Axioplan epifluorescence microscope (Carl Zeiss, Le Pecq, France) under 40× magnification. Quantifications were performed bilaterally every 100 μm sections through the ArcN (2.3–1.6 mm anterior to the interaural line) and ventromedial nucleus (VMH, 2.5–2.3 mm anterior to the interaural line) of the hypothalamus and in the NTS (3.7 mm posterior to the interaural line) and AP (3.7 mm posterior to the interaural line) of the brainstem (Franklin and Paxinos, 1997).

NPY neurons were visualized using GFP fluorescence. GFP-positive cell bodies expressing cFos were quantified unilaterally under 40× magnification using a confocal SP5 microscope (Leica, Wetzlar, Germany). Co-localizations were determined with the Image-J software (http://rsbweb.nih.gov/ij/) on series of continuous optical sections with 0.5 μm increment along the *z*-axis of the section.

### Statistical analyses

Values are given as mean ± SEM. Statistical analyses were performed using ANOVA, repeated measures ANOVA followed by Fisher PLSD *post hoc* test using the Statview software (SAS Institute Inc., Cary, NC, USA).

## Results

### Effect of GHS-R1a agonists and antagonists on feeding behavior

**Figure 1:**
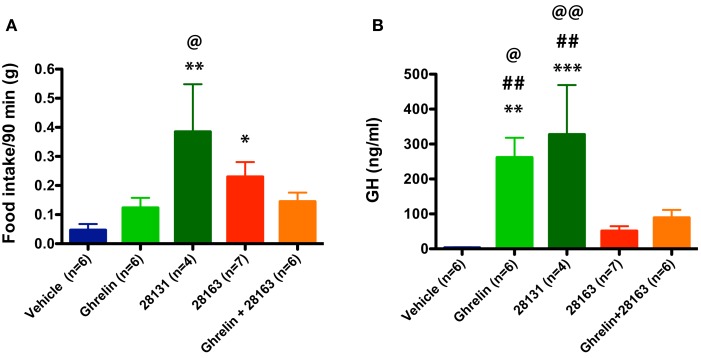
**Effect of native ghrelin and BIM compounds on cumulative food intake and GH secretion measured during the light cycle in mice**. Data represent mean ± SEM. **(A)** Mean cumulative food intake (90 min) after ip injection of native ghrelin (30 nmol), BIM-28131 (30 nmol), BIM-28163 (150 nmol), and native ghrelin (30 nmol) co-administered with BIM-28163 (150 nmol). ANOVA shows an effect of treatment on 90 min food intake: *p < 0.05 BIM-28163 vs. vehicle, **P < 0.01 BIM-28131 vs. vehicle, and native ghrelin, ^@^*P* < 0.05 BIM-28131 vs. native ghrelin + BIM-28163, Fisher PLSD *post hoc* test. **(B)** GH secretion measured by tail bleeding 15 min after ip injection of BIM compounds in the same animals. ANOVA shows an effect of treatment on GH levels: **p < 0.01 native ghrelin vs. vehicle, ***P < 0.001 BIM-28131 vs. vehicle, ^@^*P* < 0.05 native ghrelin vs. native ghrelin + BIM-28163, ^@@^*P* < 0.01 BIM-28131 vs. native ghrelin + BIM-28163, ^##^*P* < 0.01 native ghrelin or BIM-28131 vs. BIM-28163, Fisher PLSD *post hoc* test.

**Figure 2:**
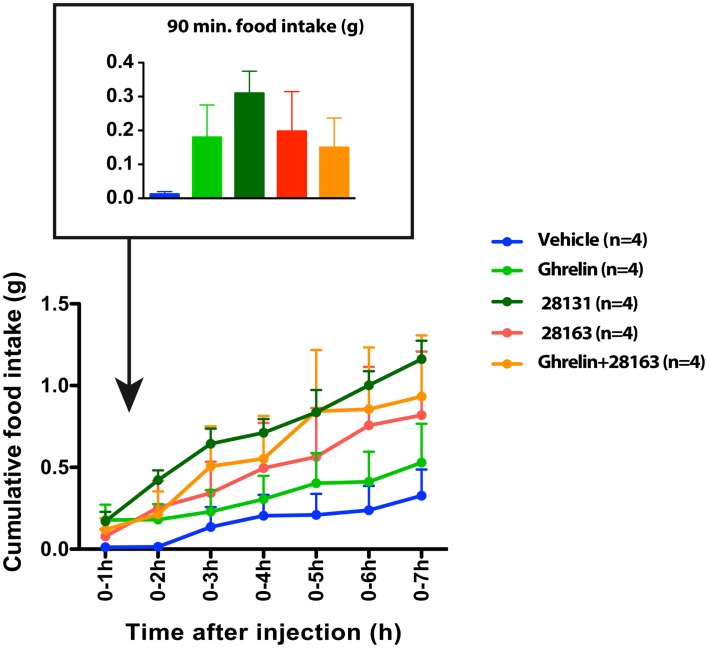
**Effect of native ghrelin and BIM compounds on cumulative food intake measured with the automated feeding station in mice**. Mean cumulative food intake (0–7 h) after ip injection of native ghrelin (30 nmol), BIM-28131 (30 nmol), BIM-28163 (150 nmol), and native ghrelin (30 nmol) co-administered with BIM-28163 (150 nmol). Data represent mean ± SEM. Repeated measures ANOVA over time (7 h) and across treatments shows an interaction between time and treatment on cumulative food intake. *P* = 0.06 BIM-28131 vs. vehicle, Fisher PLSD *post-hoc* test.

Feeding was monitored after intraperitoneal injections of native ghrelin (30 nmol), BIM-28163 (150 nmol), BIM-28131 (30 nmol), or after co-administration of native ghrelin + BIM-28163 in the early light phase. In a first subset of mice, feeding was monitored manually after injection of the treatments in randomly assigned groups of animals. Ninety minutes after the injections, a significant effect of treatment on food consumption was observed (Figure 1A). Although native ghrelin increased food intake by twofold, the effect of this compound was not significant. Only BIM-28131 and BIM-28163 increased food consumption significantly with BIM-28131 being three times more effective and BIM-28163 two times more effective than ghrelin, respectively. Food intake was identical after co-administration of BIM-28163 and native ghrelin or after administration of ghrelin alone. Automated feeding stations were also used to monitor cumulative food intake and meal pattern during 7 h following the injection in a second set of mice: animals received each treatment randomly in a cross-over designed manner (Figure 2 and Table 1). When data were analyzed as repeated measures in the same mouse and over time, there was a significant interaction between time and treatment. BIM-28131 was the only compound to stimulate appetite even in mice that did not respond well to ghrelin. Increased food consumption in this group was associated with a tendency to increased meal number, total meal size, and total meal duration (Table 1).

**Table 1:** **Effect of native ghrelin and BIM compounds on meal pattern measured with the automated feeding station in mice**.

	Meal number *n* = 3	Total meal size (g) *n* = 4	Total meal duration (min) *n* = 4
Vehicle	1.25 ± 0.75	0.23 ± 0.15	7.50 ± 4.97
Ghrelin	3.00 ± 1.08	0.41 ± 0.17	15.50 ± 9.90
BIM-28131	4.00 ± 0.41	0.82 ± 0.10	31.50 ± 4.99
BIM-28163	3.50 ± 2.02	0.92 ± 0.57	26.75 ± 14.73
Ghrelin + BIM-28163	3.50 ± 1.04	0.76 ± 0.29	27.75 ± 12.37

*Meal number, total meal size, and total meal duration measured (0–7 h) after ip injection of native ghrelin (30 nmol), BIM-28131 (30 nmol), BIM-28163 (150 nmol), and native ghrelin (30 nmol) co-administered with BIM-28163 (150 nmol). Data represent mean ± SEM*.

### Effect of GHS-R1a agonists and antagonists on GH secretion

GH plasma levels were monitored 15 min following the injections in the same animals used to monitor food intake manually (Figure 1B). Native ghrelin and BIM-28131 equally stimulated GH secretion whereas GH secretion was not increased after injection of BIM-28163 alone. In contrast to feeding data, BIM-28163 antagonized ghrelin-induced GH secretion.

### Effect of GHS-R1a agonists and antagonists on cFos activation in the hypothalamic ArcN

**Figure 3:**
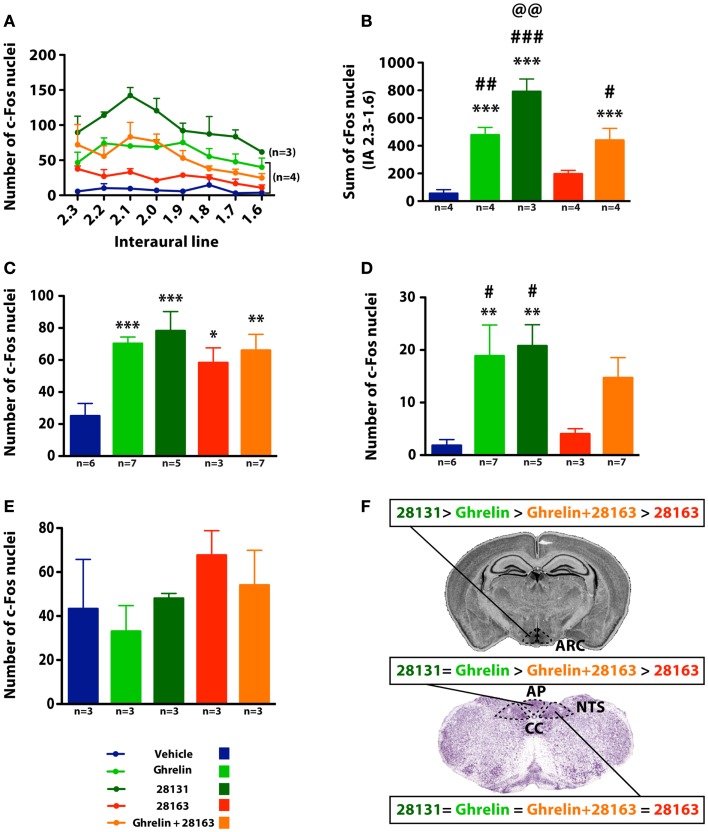
**Effect of native ghrelin and BIM compounds on cFos immunoreactivity in mice in the ArcN, AP, NTS, and VMH**. **(A)** Number of cFos immunoreactive nuclei and **(B)** sum of cFos immunoreactive nuclei along the rostro-caudal extent of the ArcN (2.3–1.6 mm anterior to the interaural line). Data represent mean ± SEM. **(A)** Repeated measures ANOVA over the rostro-caudal extent of the ArcN shows an effect of treatment on the number of cFos-positive nuclei: *P* < 0.0001 native ghrelin vs. Vehicle, BIM-28163, and BIM-28131, *P* < 0.0001 BIM-28131 vs. all other treatments, *P* < 0.0001 BIM-28163 vs. all other treatments except vehicle, *P* < 0.01 BIM-28163 vs. vehicle, *P* < 0.0001 native ghrelin + BIM-28163 vs. vehicle, BIM-28163, and BIM-28131, Fisher PLSD *post hoc* test. **(B)** ***P < 0.001 vs. vehicle, ^#^*P* < 0.05 vs. BIM-28163, ^##^*P* < 0.01 vs. BIM-28163, ^###^*P* < 0.001 vs. BIM-28163, ^@@^*P* < 0.01 vs. ghrelin + BIM-28163, Fisher PLSD *post hoc* test. **(C,D)** Number of cFos immunoreactive nuclei in the NTS and AP (3.7 mm posterior to the interaural line). Data represent mean ± SEM. ANOVA shows an effect of treatment on the number of cFos nuclei in the NTS and AP. *P < 0.05, **P < 0.01 and ***P < 0.001 vs. vehicle, ^#^*P* < 0.05 vs. 28163, Fisher PLSD *post hoc* test. **(E)** Number of cFos immunoreactive nuclei in the VMH (2.5–2.3 mm anterior to the interaural line). Data represent mean ± SEM. No significant effect of treatments is observed in the VMH. **(F)** Summary of the effect of the different BIM compounds on cFos activation in the ArcN, NTS, and AP. Cc, central canal.

cFos activation was monitored after intraperitoneal injections of native ghrelin (30 nmol), BIM-28163 (150 nmol), BIM-28131 (30 nmol), or after co-administration of native ghrelin + BIM-28163 in the early light phase. Repeated measures ANOVA over the rostro-caudal extent of the ArcN showed an effect of treatment on cFos immunoreactive nuclei (Figure 3A). All treatment groups were significantly elevated compared to the vehicle-treated group, except BIM-28163 which had a modest effect (Figure 3B). Injection of native ghrelin induced cFos activation and this was not antagonized by co-administration of BIM-28163. Administration of BIM-28131 induced a more pronounced activation than all other treatments. Thus the efficiency of activation in the ArcN was BIM-28131 > native ghrelin > native ghrelin + BIM-28163 > BIM-28163. Differences were greater around 2.1–2.0 mm anterior to the interaural line where most NPY neurons are localized.

### Effect of GHS-R1a agonists and antagonists on cFos activation in the NTS and AP of the brainstem

cFos activation was also observed in the AP and nucleus tractus solitarius (NTS) (Figures 3C,D). In the NTS, in contrast to the ArcN, all treatments significantly increased the number of cFos-immunoreactive cells. BIM-28131 stimulated cFos as efficiently as native ghrelin. BIM-28163 had an activity *per se* but was inefficient in antagonizing ghrelin-induced cFos. In the AP, the compounds had different activities. Native ghrelin and BIM-28131 had the same efficiency in activating cFos, but BIM-28163 was ineffective.

### Effect of GHS-R1a agonists and antagonists on cFos activation in the hypothalamic VMH

In a separate group of animals, cFos activation was quantified in the VMH (Interaural line: −2.5, −2.3). Due to the high level of cFos activation and high variability in vehicle-treated animals, statistical differences were not observed between treatments (Figure 3E). The number of cFos nuclei in vehicle, ghrelin, and BIM-28131-treated animals were identical; however, in mice treated with BIM-28163, the number of cFos nuclei was twice as elevated as in ghrelin-treated mice.

### Effect of GHS-R1a agonists and antagonists on cFos activation in NPY neurons

**Figure 4:**
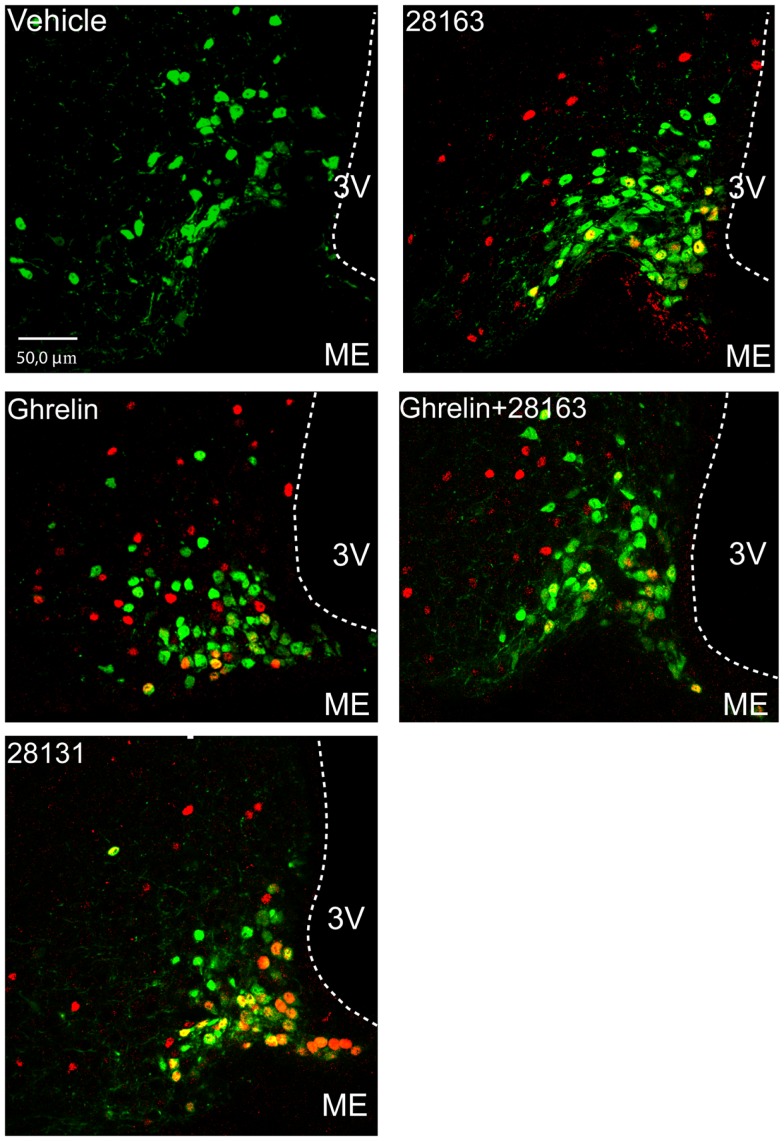
**Effect of native ghrelin and BIM compounds on cFos immunoreactivity in ArcN NPY neurons in NPY-Renilla GFP mice**. Representative confocal microphotographs showing GFP neurons (green), cFos nuclei (red), and the merge of both signals (yellow/orange) in coronal sections of the ArcN at approximately 2.1–1.9 mm anterior to the interaural line. Data represent mean ± SEM. Scale bar represents 50 μm in the ArcN. 3V, third ventricle; ME, median eminence.

To measure cFos immunoreactivity in NPY neurons, we used NPY-GFP mice. All treatments induced cFos activation in NPY neurons as compared with vehicle-treated mice (Figure 4 and Table 2). Treatments did not modify the number of GFP-positive neurons. BIM-28131 and native ghrelin co-administered with BIM-28163 induced the greatest activation of NPY neurons with more than 20% of NPY cells activated, whereas native ghrelin and BIM-28163 alone induced cFos in less than 15% of NPY neurons (Table 2).

**Table 2:** **Effect of native ghrelin and BIM compounds on the number of GFP-positive cells, number of cFos-positive nuclei, number, and percentage of GFP-positive cells expressing cFos protein in the ArcN in NPY-Renilla GFP mice**.

	GFP-positive cells	cFos-positive nuclei	GFP-positive cells expressing cFos	% GFP-positive cells expressing cFos
Vehicle (*n* = 5)	78.2 ± 10.5	6.0 ± 2.1	1.0 ± 0.4	1.5 ± 0.7
Ghrelin (*n* = 4)	106.0 ± 9.5	35.7 ± 11.4*	14.8 ± 5.8	13.9 ± 4.9
BIM-28131 (*n* = 4)	84.5 ± 9.4	43.5 ± 7.6**	18.0 ± 4.9*	20.7 ± 3.9**
BIM-28163 (*n* = 4)	102.5 ± 14.9	24.2 ± 7.5	11.5 ± 4.1	11.9 ± 3.7
Ghrelin + BIM-28163 (*n* = 4)	81.2 ± 23.1	30.2 ± 8.6*	16.0 ± 7.7*	23.4 ± 7.5**

*Coronal sections of the ArcN at approximately 2.0–1.9 mm anterior to the interaural line were quantified unilaterally. Data represent mean ± SEM*.

*P < 0.05 and **P < 0.01 vs. vehicle.

## Discussion

The present study demonstrates that two GHS-R1a synthetic ligands, BIM-28163 and BIM-28131, are powerful stimulators of appetite in mice, acting through pathways and brain regions that are distinct from the ones activated by ghrelin (Figure 3F and Table 3).

**Table 3:** **Summary of the effects of the different BIM compounds on GH secretion, food intake, and cFos activity in the ArcN, NTS, and AP after intraperitoneal administration in the mouse**.

	Food intake	GH secretion	cfos ArcN	cfos AP	cfos NTS	cfos VMH
Ghrelin	±	+++	++	+++	+++	=
28131	+++	+++	+++	+++	+++	=
28163	++	=	±	±	+++	=
Ghrelin + 28163	No antagonistic action	Antagonistic action	No antagonistic action	No antagonistic action	No antagonistic action	=

*Food intake and cFos measured 90 min and GH 15 min post-injection*.

BIM-28163 is a full GHS-R1a antagonist as it has no intrinsic activity at the GHS-R1a, and can fully block the ability of ghrelin to activate the GHS-R1a, as well as block ghrelin-induced GH release, both *in vitro* and *in vivo* (Halem et al., 2004). It is thus a pharmacological tool to dissect the role of endogenous ghrelin. However, whereas previous investigations in rats with this GHS-R1a antagonist revealed a role of the endogenous ligand in amplifying GH pulsatile pattern, blocking GHS-R1a fails to inhibit food intake (Zizzari et al., 2005), and BIM-28163 even stimulates appetite and weight gain after daily treatment in rats (Halem et al., 2004, 2005). In the current study performed in mice, we also demonstrate that acute injection of BIM-28163 increases food consumption and show for the first time that the compound induces cFos in two brain regions involved in the control of appetite, the hypothalamus and brainstem. The feeding effect of BIM-28163 is observed as early as 15–30 min after its injection (i.e., same time-course as native ghrelin) (data not shown).

It was previously described in the rat that intraperitoneal (ip) injection of native ghrelin in the early light phase induces feeding and cFos activation in the ArcN, NTS, and AP (Hewson and Dickson, 2000; Lawrence et al., 2002; Takayama et al., 2007). A dose of 30 nmol/30 g body weight was chosen here based on a published study showing that this dose stimulates food intake in mice (Zizzari et al., 2007). Although ghrelin was very potent in activating cFos in the ArcN, NTS, and AP in the present study, its effects on feeding did not reach statistical significance. It may be due to the fact that different sets of animals were used for feeding experiments and cFos experiments. Another possible interpretation is that cFos activation in these nuclei is required but not alone sufficient to induce an effect on appetite. We indeed recently observed that after ip injection of ghrelin, animals can be subdivided into two groups: high and low responders, suggesting an interindividual variability in the feeding effects of ghrelin (Hassouna et al., 2012b).

BIM-28131 is a super-agonist with regard to food intake in rats (Strassburg et al., 2008; Palus et al., 2011). It is also very potent to stimulate both appetite and cFos in the current study even in mice that were not responsive to ghrelin in the cross-over designed study. Differences in stability between native ghrelin and BIM-28131 would partly explain the differential activities of these two compounds. Indeed, in addition to a fivefold higher affinity than native ghrelin in binding to the GHS-R1a and a 10-fold increased potency in activating the receptor, BIM-28131 has a 10-fold greater circulating half-life. However, BIM-28131 is comparable to ghrelin with regard to stimulating GH secretion. The equal effect on GH release may be possible because the action of the compounds may be partly relayed at the pituitary level and the time of blood sampling occurred after a relatively short time (15 min) whereas the food intake was measured over 90 min or greater. By analyzing the feeding response every 15 min following the injection, BIM-28131 did not have a stronger feeding effect over native ghrelin at 15–30 min post-injection (data not shown) but from 60 min following the injection. Thus, BIM-28131 is more powerful than ghrelin in stimulating appetite at equimolar doses, and is also more powerful in activating cFos in the ArcN, but not in the NTS and AP, suggesting that the activity of this super-agonist is mostly mediated in the ArcN. This is further substantiated by cFos activation in orexigenic NPY ArcN neurons in BIM-28131-treated mice as compared with ghrelin-treated ones. NPY neurons are well-known targets for ghrelin actions (Tannenbaum et al., 2003; Chen et al., 2004) and induction of cFos in NPY neurons in rats was previously demonstrated with other ghrelin agonists (Dickson and Luckman, 1997).

In the current study, BIM-28163 activates several brain nuclei. The intensity of activation seems to be different depending on the region and relative to that of native ghrelin. BIM-28163 appears to have greater activity in the NTS than in the ArcN and AP, two structures outside the blood brain barrier (BBB). Indeed, in the NTS, BIM-28163 is as potent as both native ghrelin and BIM-28131 in inducing cFos with more than a twofold increase as compared with saline treated animals although the effect does not reach statistical significance. In the ArcN, however, BIM-28163 is not as effective as ghrelin in stimulating cFos, and is much less active than BIM-28131. This suggests that BIM-28163 uses alternative pathways other than through these BBB free structures to relay its orexigenic actions. These data are consistent with previous reports in rats showing that BIM-28163 selectively activates cFos in the DMH after icv injections, whereas, in the ArcN, it acts exclusively as an antagonist by blocking ghrelin-induced cFos activation without any intrinsic effect (Halem et al., 2004, 2005). Our data here slightly differ from the above studies in rats because BIM-28163 still activates cFos in the ArcN, although to a much lesser extent than ghrelin. In addition, we were not able to observe any antagonistic actions of BIM-28163 either on food intake or cFos activity when co-administered with ghrelin. Differences may be due to the species studied (mouse vs. rat), to the mode of administration (ip vs. icv), as well as the dose injected (5 nmol/g ip vs. 1.5 nmol/rat icv).

Differences in antagonizing ghrelin-induced food consumption or cFos may be due to the fact that BIM-28163 was injected at a fivefold higher dose than ghrelin instead of a 10-fold higher dose (IC50 for BIM-28163 at GHS-R1a is 10-fold higher than for native ghrelin). However, the antagonistic effect of BIM-28163 on ghrelin-induced cFos activity in rats was still observed when BIM-28163 was administered at a fivefold dose (Halem et al., 2005). The recent report by Costantini et al. (2011) shows that another novel and selective GHS-R1a antagonist with no partial agonist activity, GSK1614343 was also not able to antagonize ghrelin-induced food intake at a dose of 10 mg/kg.

Concerning the VMH, a high level of activation and high interindividual variability in vehicle-treated animals are observed. Consequently, ghrelin seems to have no stimulatory action in this nucleus. This is consistent with other data in rats showing that ghrelin does not activate cFos in the VMH (Lawrence et al., 2002). In contrast, the number of cFos-positive nuclei after treatment with BIM-28163 is almost twofold higher than in the ghrelin-treated group.

Within the hypothalamus, the ArcN is one of the main targets of peripheral signals, such as leptin and ghrelin, which relay information about energy stores and/or nutritional status. The efficiency of the compounds used in this study in activating the ArcN is BIM-28131 > native ghrelin > native ghrelin + BIM-28163 > BIM-28163. The GHS-R1a has been shown to be co-expressed with several neuropeptides in the ArcN. It is expressed on the orexigenic NPY and GHRH neurons (Willesen et al., 1999; Tannenbaum et al., 2003) and these populations of neurons relay ghrelin orexigenic and GH-releasing actions in rats (Tannenbaum et al., 2003; Chen et al., 2004). In mice, ghrelin also induces cFos in NPY-expressing neurons (Wang et al., 2002). To determine whether BIM-28163 and BIM-28131 orexigenic actions could be partly mediated through NPY neurons as is the case for ghrelin, we investigated the effect of these compounds in NPY-GFP mice. Indeed, ip administration of 30 nmol ghrelin during the light cycle induced cFos activation in approximately 26% of NPY neurons (Hassouna et al., 2012a,b). Here we observed that about 15% of NPY-positive cells were activated after BIM-28163 or native ghrelin administration whereas about 20% were activated after BIM-28131 treatment. The orexigenic action of BIM-28163 could be partly mediated through an activation of ArcN NPY neurons. Orexigenic actions of ghrelin may also be mediated by a reduced activity in POMC cells (Cowley et al., 2003). Modified activity of the anorexigenic POMC neurons after treatment with GHS-R1a compounds can thus not be excluded. Orexigenic actions of GSK1614343, another GHS-R1a antagonist, was accompanied by a reduced expression of POMC in the ArcN after chronic treatment with the compound (Costantini et al., 2011).

Although the majority of studies demonstrated that acylation is essential for ghrelin feeding activities (Inhoff et al., 2009), one study showed that desacyl ghrelin was able to stimulate food intake after intracerebroventricular administration by a mechanisms independent of the GHS-R1a (Toshinai et al., 2006). Interestingly, feeding effects of desacyl ghrelin was more pronounced in GHS-R1a deficient mice. Thus it can be postulated that blocking the GHS-R1a with the antagonist may allow desacyl to stimulate feeding through a GHS-R1a independent pathway. Whereas a distinct receptor from the known GHS-R1a would possibly mediate the orexigenic actions of BIM-28163, we can not exclude that these effects are also dependent on the GHS-R1a. Indeed, a recent study using the antagonist, GSK1614343, showed that the appetite-mediated action of this compound was abolished in GHS-R null mice (Costantini et al., 2011), suggesting that the orexigenic effects of GSK1614343 is relayed by GHS-R1a or that the ghrelin receptor may be needed. GHS-R1a is associated with multiple signal transduction pathways (Carreira et al., 2004; Holst et al., 2005) and it is possible that BIM-28163 could activate a specific pathway on the GHS-R1a that is independent from the one mediating GH-releasing activities. In addition, the formation of heterodimers between the GHS-R1a and other receptors has been evidenced (Kern et al., 2012), and raises the question as to whether the orexigenic actions of BIM-28163 could be mediated through interaction with a GHS-R1a dimer that differs from the GHS-R1a receptor form that regulates GH secretion. BIM-28163 could possibly interact with a receptor that needs to dimerize with the GHS-R1a.

The present study demonstrates that two GHS-R1a synthetic ligands, BIM-28163, and BIM-28131, are powerful stimulators of appetite in mice, acting through pathways and brain regions that are distinct from those activated by ghrelin.

In conclusion, utilization of synthetic GHS-R1a ligands, such as BIM-28163 and BIM-28131 that are powerful stimulators of appetite and act through pathways that are distinct from those activated by ghrelin, even in situations when ghrelin seems modestly effective, can have important clinical implications, in conditions such as cachexia or anorexia [*see other chapter in the same issue: Ghrelin: Central and Peripheral Implications in Anorexia Nervosa* (Mequinion et al., 2012)]. BIM-28131 was previously demonstrated to be very efficient in a rat heart failure model of cachexia (Strassburg et al., 2008; Palus et al., 2011). In addition, observations that BIM-28163 is able to selectively stimulate feeding and increase weight gain without altering GH secretion may suggest the possibility of treating pathologies in which hyper-activity of the GH/IGF-1 axis may be deleterious. A better understanding of the molecular pathways activated by these compounds will be useful for devising future therapeutic applications.

## Conflict of Interest Statement

The authors declare that the research was conducted in the absence of any commercial or financial relationships that could be construed as a potential conflict of interest.
